# Tceal7 is a BRG1-regulated target of calcineurin signaling that promotes myoblast differentiation

**DOI:** 10.1371/journal.pone.0358038

**Published:** 2026-09-16

**Authors:** Sandhya Yadav, Tapan Sharma, Monserrat Olea-Flores, Teresita Padilla-Benavides, Anthony N. Imbalzano

**Affiliations:** 1 Department of Biochemistry and Molecular Biotechnology, University of Massachusetts Chan Medical School, Worcester, Massachusetts, United States of America; 2 Department of Molecular Biology and Biochemistry, Wesleyan University, Middletown, Connecticut, United States of America; University of Milan, ITALY

## Abstract

The transcriptional control of skeletal muscle differentiation requires the coordinated activity of lineage-defining transcription factors, signal-responsive regulators, chromatin modifiers, and ATP-dependent chromatin remodeling enzymes. Here, we identify TCEAL7, a member of the X-linked, poorly characterized TCEAL family of proteins, as a direct downstream target of BRG1-containing mammalian SWI/SNF (mSWI/SNF) complexes and calcineurin signaling during myoblast differentiation. Analyses of previously published datasets showed that pharmacological inhibition of mSWI/SNF bromodomains or knockdown of the BRG1 ATPase, but not knockdown of the homologue BRM ATPase, significantly reduced *Tceal7* expression in differentiating C2C12 myoblasts. We demonstrate that BRG1 occupancy at the *Tceal7* promoter increased during differentiation, paralleling the induction of *Tceal7* expression and nuclear accumulation of TCEAL7 protein. BRG1 functions in part by integrating calcium-dependent cues via the phosphatase calcineurin (Cn); we also determined that Cn knockdown or pharmacological inhibition of Cn suppressed *Tceal7* expression and impaired myoblast differentiation. The data suggest that both BRG1-driven chromatin remodeling and Cn signaling converge on *Tceal7* regulation. Functionally, *Tceal7* knockdown altered cell proliferation and disrupted myoblast differentiation, at least in part due to reduced expression of *Myogenin*, which encodes a transcription factor that is an essential differentiation determinant. RNA-seq analysis revealed broad dysregulation of myogenic, metabolic, and cell-cycle gene programs in *Tceal7*-deficient cells, including changes in cyclin-dependent kinase-regulated pathways consistent with prior reports linking TCEAL7 to cell-cycle control. Together, these findings identify TCEAL7 as a necessary component of the myogenic regulatory network whose expression is controlled by BRG1-dependent chromatin remodeling and Cn activity.

## Introduction

The TCEAL family of proteins is encoded by a cluster of X chromosome-linked genes that are evolutionarily conserved in mammals [1,2]. The first member of the family identified, now called TCEAL1 [3], was reported to share homology with the transcription elongation factor A (TCEA), also known as TFIIS [4–7]. Specifically, TCEAL1 shared limited homology with the amino acid sequences identified as interacting with RNA polymerase II and was reported to have a zinc (Zn) finger-“like” domain resembling the Zn finger existing in TFIIS [3]. Other TCEAL proteins and the genes encoding them were identified and named based on their homology to TCEAL1. There are nine TCEAL proteins in humans and seven in mice [2]. The TCEAL family members are presumed to play a role in transcription elongation or, since TFIIS was later shown to also be involved in transcription initiation by RNA polymerase II [5,7], transcription regulation in general. However, to our knowledge, there are no reports demonstrating that any of the TCEAL proteins bind Zn, bind RNA polymerase II, or function directly in transcription. We recently compared the known structure of TFIIS to the structures of each TCEAL protein as predicted by Alphafold2 [8,9] and proposed that it is likely that none of the TCEAL proteins bind either Zn or RNA polymerase II [10]. Functionally, TCEAL protein and/or mRNA levels frequently have been identified as mis-regulated, both positively and negatively, in many types of cancer [10]. Several TCEAL proteins have links to protein de-ubiquitination [11–13], and most are predicted to be phosphoproteins [10]. However, a unifying function, if one exists, remains unknown.

Mammalian SWI/SNF (mSWI/SNF) enzymes are ATP-dependent chromatin remodelers [14–16]. These enzymes are multi-subunit complexes with significant diversity in assembly, resulting in the formation of many different enzyme complexes [17,18]. However, biochemical studies of enzyme assembly revealed that there are three main sub-families, each characterized by unique subunits [19]. The enzymes alter nucleosome structure to facilitate access to DNA during transcription, replication, recombination and repair [20–23]. Consequently, the mSWI/SNF enzymes are critical for development and for many normal cellular processes. Not surprisingly, mis-regulation or mutation/deletion of different subunits are associated with many types of cancer [24–26], and it has been reported that ~20% of all human cancers contain mis-regulated or mutated mSWI/SNF enzyme subunits [26].

mSWI/SNF chromatin remodelers have been shown to be critical for the differentiation of myoblasts in culture and during adult skeletal muscle differentiation and function [27–33]. Altered expression or mutation of mSWI/SNF enzyme subunits results in mis-regulation of genes essential for cell cycle regulation proteins [34,35], differentiation [27,36,37], and those of the Wnt signaling pathway [38–40], which is critical for myogenesis [41,42].

Our group has reported the deleterious effects of a drug called PFI-3 on myoblast differentiation [32]. PFI-3 is a bromodomain inhibitor that specifically binds to bromodomains in three distinct mSWI/SNF subunits (BAF180 and the mutually exclusive ATPases, BRM and BRG1), but no other proteins [43,44]. We have also examined the effects of siRNA treatment targeting BRG1, BRM or both [45]. PFI-3 treatment and knockdown of BRG1, BRM or both inhibited myoblast differentiation in culture and PFI-3 inhibited in adult myogenesis after injury *in vivo* [32,45]. Among the genes that were sensitive to PFI-3 treatment and BRG1, but not BRM, siRNA was a TCEAL gene, *Tceal7*.

TCEAL7 was of interest because it has been implicated in myogenesis [46–50]. The *Tceal7* gene was differentially expressed during skeletal muscle regeneration following injury in adult mice, while *Tceal7* mRNA was expressed only in skeletal muscle during mouse embryogenesis [47]. *Tceal7* gene expression changes were also identified in skeletal muscle using different mouse disease models [50,51] and during *in vitro* mouse myoblast differentiation [52]. Studies of the regulation of *Tceal7* gene expression in skeletal muscle originally utilized transgenic mice to identify a 0.7 kb portion of the sequences upstream of the *Tceal7* transcription start site (TSS) that could recapitulate expression in developing embryonic skeletal muscle [47]. Mutation of the E boxes in this sequence, which are binding sites for the myogenic regulatory factor (MRF) family of basic helix-loop-helix transcription factors that includes MyoD1, myogenin, Mrf4, and Myf5 [53], resulted in loss or reduction of transgene expression [47,49]. Each member of the MRF family stimulated *Tceal7* expression and at least one of the MRFs, MyoD1, could bind to the *Tceal7* E boxes [47]. Subsequent work showed that additional transcriptional regulators known to impact myogenic gene expression, MEF2C and CREB1, could cooperate with MyoD1 to regulate *Tceal7* expression [49].

Functionally, myoblasts overexpressing *Tceal7* showed enhanced differentiation capacity. The expression of several cell cycle regulators was surveyed; the only tested mRNA to show a change was *Cdkn1b*, which encodes p27 and which was elevated [48]. p27 is a cyclin dependent kinase inhibitor that functions to reduce cell proliferation [54], which likely at least partially explains the effect of *Tceal7* overexpression. Transgenic mice expressing *Tceal7* in skeletal muscle after the initiation of skeletal muscle development did not impact embryogenesis or post-natal growth for the first three weeks [54]. However, the overall size and body weight was altered subsequently, at least in part due to reduced cross-sectional area of examined skeletal muscles [54]. Additionally, direct interaction between TCEAL7 and CDK1 was observed [54]. CDK1 is a cyclin-dependent kinase that, when coupled with a partner cyclin, phosphorylates target proteins involved in protein synthesis [55]. The authors proposed that the TCEAL7 protein repressed CDK1 activity, thereby negatively impacting protein synthesis and affecting myofiber growth [54]. TCEAL7 may therefore impact both myoblast differentiation and myofiber growth. Another study showed that knockdown of either *Tceal7* or *Tceal5* did not affect differentiation in culture but simultaneous knockdown of both did [46].

Here, we show that *Tceal7* expression is controlled by BRG1-containing SWI/SNF chromatin-remodeling complexes. *Tceal7* is robustly induced during myogenic differentiation, localizes to the nucleus, and is essential for proper *Myogenin* expression, myotube fusion, and activation of myogenic transcriptional networks. shRNA-mediated *Tceal7* knockdown severely impaired myoblast differentiation and broadly reduced the expression of myogenic genes, while inducing signaling pathways that likely hinder, rather than support, normal myoblast differentiation. Together, our findings support a model where *Tceal7* is a critical regulator of the transcriptional and signaling programs that drive myoblast differentiation.

## Materials and methods

### Antibodies

The primary antibodies against BRG1 that were used for western blot and chromatin IP (sc-17796; 1:1,000 and sc-17796 G-7X, 3-4 μL per μg of chromatin, respectively), against vinculin (sc-25336; 1:1000), and against Lamin β1 (sc-56144; 1:10,000) were purchased from Santa Cruz Biotechnologies. Myosin Heavy Chain monoclonal antibody (MF20, deposited by D. A. Fischman) was purchased from the Developmental Studies Hybridoma Bank, University of Iowa. The calcineurin antibody was from Cell Signaling Technology, Inc (2614; 1:1,000). The TCEAL7 antibody was from MyBioSource (MBS9419677; 1:1000). The secondary antibodies used were goat anti-rabbit and -mouse coupled to HRP (31460, 31430 respectively; 1:5,000) and were acquired from Thermo Fisher Scientific.

### Mammalian cell culture

C2C12 cells are a karyotypically abnormal subclone of a spontaneously immortalized mouse skeletal myoblast line widely used to model myoblast proliferation and differentiation [56]. C2C12 cells were obtained from ATCC (Manassas, VA) and maintained at sub-confluent densities in proliferation medium consisting of Dulbecco’s Modified Eagle Medium (DMEM; 11965118, ThermoFisher Scientific) supplemented with 10% fetal bovine serum (FBS) and 1% penicillin-streptomycin in a humidified incubator at 37°C with 5% CO_2_. C2C12 differentiation was initiated when cultures reached approximately 80% confluence. Cells were switched to differentiation medium containing DMEM supplemented with 2% horse serum, 1% insulin-transferrin-selenium A (Invitrogen), and 1% penicillin-streptomycin. Differentiated myoblasts were collected at the time points indicated in the figure legends and processed for further analyses. PFI-3 (15267; Cayman Chemicals) was added to cell culture media at a final concentration of 50 μM at the time of induction of differentiation [32].

### siRNA transfection of C2C12 myoblasts

The following siRNA oligos were purchased from Dharmacon Horizon Discovery Ltd., United States. siRNA against *Brg1* (siGENOME mouse *Smarca4* pool #M-041135-01-0020, 50nM; siGENOME mouse *Smarca4* #D-041135-03-0050, 25nM, referred to as si*Brg1*-A; and siGENOME mouse *Smarca4* #D-041135-04-0050, 25nM, referred to as si*Brg1*-B). The non-targeting siRNA (SMARTpool ON-TARGETplus scrambled # D-001810- 10-20, 50 nM) was used as control. C2C12 cells were transfected as previously described [32] using indicated concentrations of different siRNAs and harvested at indicated times for further analysis.

### shRNA-mediated gene knockdown

shRNA-mediated knockdown used procedures and shRNAs against *CalcineurinA* and a scrambled sequence shRNA that were previously described [57]. The shRNA sequence for *Tceal7* shRNA1 was CCGGCCAGCGACTGGAAGGCAATTTCTCGAGAAATT-GCCTTCCAGTCGCTGGTTTTTG (TRCN 0000245255) and for *Tceal7* shRNA2 was CCGGAGACAATCATCTGGTAGTTTACTCGAGTAAACTACCAGATGATTGTCTTTTTTG (TRCN0000256702). Both were purchased from SigmaAldrich.

### Western blot analysis

C2C12 cell samples from three independent experiments were washed twice with PBS and scraped into 1 mL PBS using a cell lifter. Cells were pelleted by centrifugation and lysed in 500 μL RIPA buffer (50 mM Tris–HCl, pH 7.4; 150 mM NaCl; 1 mM EDTA; 1% NP-40; 0.25% sodium deoxycholate) supplemented with protease inhibitor cocktail (P8340, Sigma Aldrich). Lysates were sonicated in an ultrasonic bath for three 30 s on/off cycles at 4°C. Samples were centrifuged at 14,000 × *g* for 10 min at 4°C, and supernatants were collected. Protein concentrations were measured using the Pierce™ BCA assay (ThermoFisher Scientific). A total of 20 μg protein mixed with loading dye (5% β-mercaptoethanol, 0.02% bromophenol blue, 30% glycerol, 10% SDS, 250 mM Tris-Cl, pH 6.8) was boiled at 95°C for 10 min and resolved on 10% SDS-PAGE gels, followed by transfer to Immobilon-P PVDF membranes (Merck Millipore). Membranes were blocked in 5% non-fat milk for 30 min and incubated overnight at 4°C with primary antibodies diluted in 2% milk in PBS or TBS. After three 5-min washes in TBS with 0.1% Tween-20, membranes were incubated for 1 h at room temperature with HRP-conjugated species-specific secondary antibodies, followed by three additional washes. Bands were detected by chemiluminescence using ECL Plus (GE Healthcare) on an Amersham Imager 600. Band intensities from three independent experiments were quantified using ImageJ [58].

### Cell proliferation assays

C2C12 myoblasts were seeded at 1 × 10^4^ cells/cm^2^, and samples were collected at 24, 48, and 72 h post-plating. Cells were trypsinized, washed three times with PBS, and counted using a Cellometer Spectrum (Nexcelom Biosciences).

### RT-qPCR gene expression analysis

Total RNA was isolated from three independent biological replicates of differentiated C2C12 myoblasts using TRIzol (Invitrogen) according to the manufacturer’s instructions. cDNA was synthesized from 500 ng RNA using random primers and SuperScript III reverse transcriptase (Invitrogen). Quantitative PCR was performed with Fast SYBR Green 2X Master Mix (Applied Biosystems), and final reaction volume was adjusted to 10 μL per reaction. All qPCRs were run in QuantStudio 3 RT-PCR machine (Applied Biosystems) using primers listed in S1 Table. ΔC_T_ was calculated as the C_T_ of the target gene minus the C_T_ of the housekeeping gene (Eef1a1). ΔΔC_T_ was then obtained by subtracting the average ΔC_T_ of the control group from the ΔC_T_ of each sample. Relative expression was determined using the 2^-ΔΔCT^ method [59].

### Immunocytochemistry Analyses

C2C12 myoblasts (control and knockdown) were fixed overnight in 10% formalin-PBS at 4°C, washed with PBS, and permeabilized for 10 min in PBS with 0.2% Triton X-100. Immunocytochemistry was performed using the myosin heavy chain hybridoma supernatants. Staining was developed using the Universal ABC kit (PK-6200; Vector Laboratories, United States) and HRP DAB substrate (SK-4100; Vector Laboratories, United States) according to the manufacturer’s instructions.

### Chromatin Immunoprecipitation (ChIP) Assays

ChIP assays were performed as previously described [57]. Briefly, differentiating C2C12 myoblasts were cross-linked with 1% formaldehyde for 10 min at room temperature and quenched with 125 mM glycine for 5 min. Cells were washed twice with ice-cold PBS containing protease inhibitors and lysed in 1 mL of ice-cold SimpleChIP ® Enzymatic Cell Lysis Buffer A (14282; Cell Signaling Technology). Nuclei were pelleted at 3000 × g, washed in buffer B (14231; Cell Signaling Technology) as recommended by the manufacturer. Chromatin was digested with 1000 U micrococcal nuclease (M0247S; New England Biolabs) for 30 min at 37°C. Reactions were stopped with 5 μL of 0.5 M EDTA. Nuclei were pelleted, resuspended in 400 μL ChIP buffer (SimpleChIP ® Chromatin IP Buffers, Cell Signaling Technology) supplemented with protease inhibitors, sonicated for 10 min (30 s on/30 s off, medium intensity) using a Bioruptor UCD-200 (Diagenode), and centrifuged at 21,000 × g for 5 min. Fragmented chromatin (200-500 bp) was confirmed by agarose gel electrophoresis.

Chromatin was incubated for 2 h at 4°C with antibodies against BRG1 or with an anti-IgG antibody as a negative control. Immunocomplexes were captured with 20 μL Dynabeads (Thermo Fisher Scientific) after overnight incubation at 4°C, washed three times with low-salt ChIP buffer and once with high-salt buffer, and eluted in 100 μL elution buffer (0.1 M NaHCO_3_, 1% SDS) for 30 min at 65°C. Samples were treated with RNase (1 μL, 0.5 mg/mL, 30 min, 37°C), then reverse-cross-linked overnight at 65°C with 6 μL 5 M NaCl and 1 μL proteinase K (1 mg/mL). DNA was purified using the ChIP DNA Clean & Concentrator kit (Zymo Research). Enriched DNA was analyzed by qPCR using SYBR Green master mix and quantified by the 2^(ΔCT sample – ΔCT IgG)^ method. The data are shown relative to the results determined for IgG controls. Primer sequences are listed in S1 Table.

### RNA-sequencing analysis

Triplicate samples of approximately 4 million C2C12 myoblasts differentiated for 72 h were harvested and RNA was isolated as described above. Total RNA was assessed for quality and concentration using the UMass Chan Molecular Biology Core Lab Fragment Analyzer. The samples demonstrated an RNA integrity number (RIN) ≥ 7 and 28S/18S ≥ 1.0. 50 micrograms of RNA for each sample were submitted to BGI Americas for library preparation and RNA-seq was performed with 1 ug of RNA using the vendor’s standard procedures. After adapter trimming, clean reads were aligned to the *mm10* reference transcriptome using HISAT2, and gene expression was quantified with featureCounts [60]. Differentially expressed genes were identified using DESeq2 [61], applying thresholds of log_2_ fold change ≥ ±0.5 and adjusted P < 0.05. Dot plots were generated in RStudio using ClusterProfiler [62]. Pathway enrichment was performed with the PANTHER database [63,64]. RNA-seq data was deposited and is available at GEO under the accession number GSE319350.

### Statistical analysis

Statistical analyses were performed using GraphPad Prism 7.0b. Statistical comparisons between two groups were performed using two-tailed Welch’s t-test. Data are presented as mean ± SD, and *P* < 0.05 was considered statistically significant.

## Results

### BRG1 is required for *Tceal7* expression during myoblast differentiation

**Fig 1:**
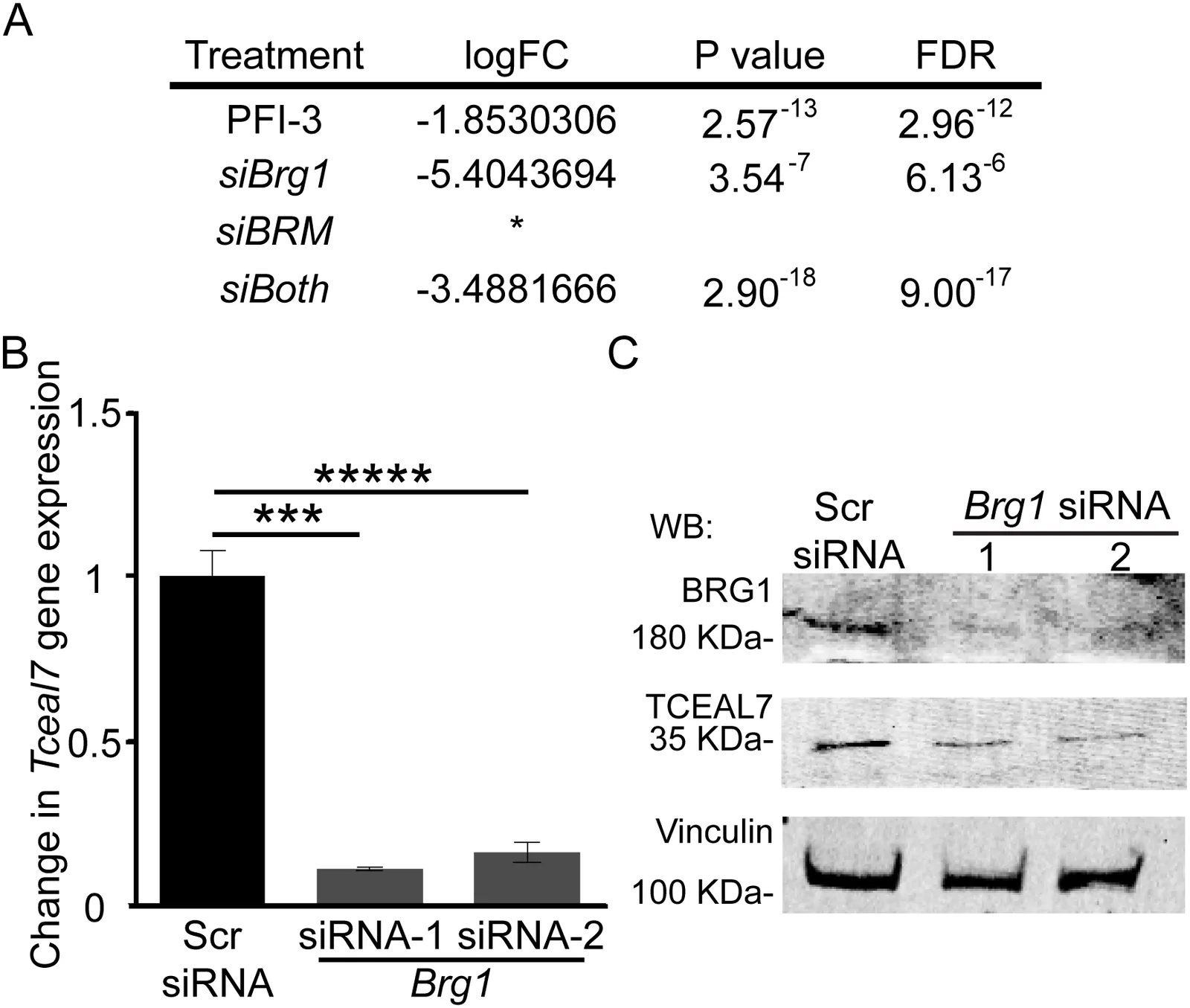
*Tceal7* expression is regulated by BRG1 but not BRM. **(A)** *Tceal7* gene expression levels in differentiating C2C12 myoblasts treated with PFI-3 (from GSE151218) or treated with siRNAs targeting *Brg1* or *Brm* (from GSE196283). The asterisk indicates the gene was not differentially expressed. **(B)** Steady state *Tceal7* mRNA expression in C2C12 cells transfected with control scrambled sequence (Scr) or one of two different *Brg1* siRNAs, normalized to *Eef1a* expression. **(C)** Western blots showing reduced TCEAL7 protein levels upon *Brg1* knockdown. Vinculin served as the loading control. Data represent 3 independent biological experiments ± SD. Two-tailed Welch’s t-test statistical analyses. ***P < 0.001; *****P < 0.00001.

We previously performed RNA-seq on differentiating C2C12 cells that had been treated with PFI-3, a bromodomain inhibitor that binds to and interferes with bromodomains on three mSWI/SNF subunits: the mutually exclusive ATPases, BRG1 and BRM, and the BAF180 subunit specific to the PBAF subfamily of mSWI/SNF complexes [32,44]. In that study, we demonstrated that the effects of PFI-3 were due to inhibition of BRG1 and BRM; BAF180 was dispensable for myoblast differentiation [32]. We also previously analyzed differentiating C2C12 cells treated with siRNA targeting *Brg1*, *Brm*, or both [45]. We noted that *Tceal7*, a gene previously implicated in skeletal muscle development [46–50], was down regulated in differentiating C2C12 cells treated with PFI-3 and siRNA targeting *Brg1*, but not in cells treated with siRNA targeting *Brm* (Fig 1A). To directly validate *Tceal7* as a BRG1 target, we performed siRNA-mediated knockdown experiments using these cells. *Brg1* knockdown markedly reduced *Tceal7* mRNA and TCEAL7 protein abundance (Fig 1B,1C). These findings demonstrate that *Tceal7* is transcriptionally controlled by BRG1 and functions as a downstream effector within BRG1-based mSWI/SNF-mediated myogenic regulatory networks.

### *Tceal7* is induced during C2C12 myoblast differentiation and its promoter is inducibly bound by BRG1 during differentiation

**Fig 2:**
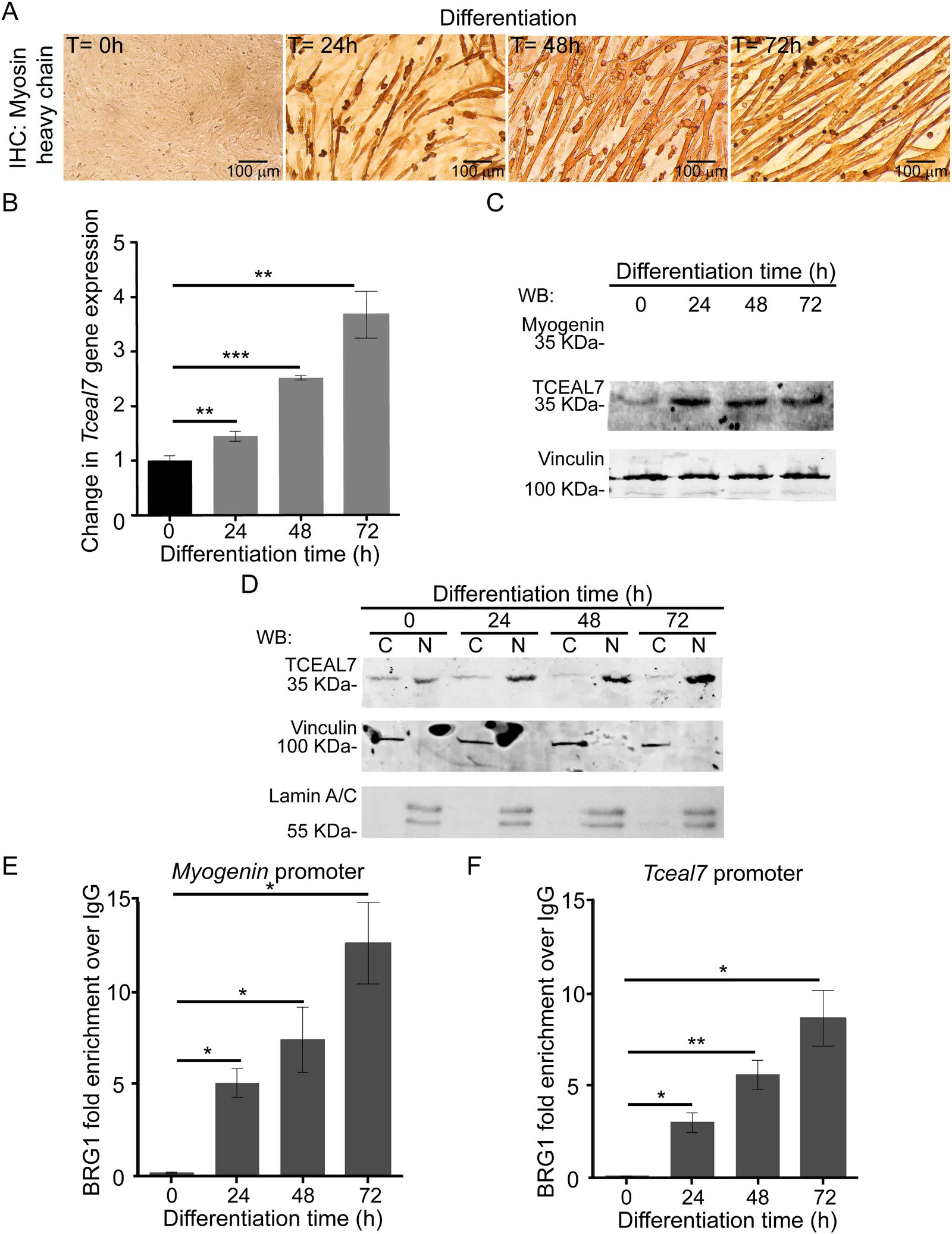
TCEAL7 is induced during C2C12 differentiation and accumulates in the nucleus and the cytoplasm; the *Tceal7* promoter is bound by BRG1. **(A)** Representative light microscopy images of myosin heavy chain staining of differentiating myoblasts at 0, 24, 48, and 72 h. **(B)** Steady state levels of *Tceal7* mRNA levels during the time course of differentiation, normalized to *Eef1a*. **(C)** Representative western blot showing TCEAL7 and myogenin protein expression over the differentiation time course; vinculin was used as the loading control. **(D)** Representative western blot analysis of TCEAL7 in cytoplasmic **(C)** and nuclear (N) fractions at indicated time points. To determine purity of fractions, vinculin was used as the cytoplasmic extract control and Lamin A/C was used as the nuclear extract control. (E-F) ChIP analysis showing BRG1 binding at the *Tceal7* **(E)** and *Myogenin* **(F)** promoters across the differentiation time course. Values were normalized to values obtained for an IgG ChIP. Data represent 3 independent biological experiments ± SD. Two-tailed Welch’s t-test statistical analyses. * P < 0.05; ** P < 0.01; ***P < 0.001.

To determine how *Tceal7* expression changes during C2C12 myoblast differentiation, we examined expression at 24 h intervals from the onset of differentiation to 72 hours post-differentiation. Immunohistochemistry of the differentiation marker myosin heavy chain (MHC) showed the transition from single C2C12 myoblasts to elongated, multi-nucleated myotubes (Fig 2A), thereby providing a visual assessment of the state of the cells at each timepoint. *Tceal7* transcript levels increased during this period (Fig 2B), and immunoblotting revealed a similar increase in the protein levels of TCEAL7 and myogenin, an early myogenic marker (Fig 2C). The increase in TCEAL7 protein level is more rapid than is the increase in *Tceal7* mRNA. This suggests the possibility of post-transcriptional regulation.

### TCEAL7 is found in both the cytoplasm and the nucleus of differentiating myoblasts

Evidence linking TCEAL7 to CDK1 function [48] and inhibition of the ability of the transcription factor NF-KB to bind DNA [65] suggests TCEAL7 is a nuclear protein, and HA-tagged TCEAL7 localizes to the nucleus [66]. Furthermore, analysis of human samples showed that TCEAL7 was present in the nucleus of normal gastric tissues as well as in differentiated gastric tumors [67].

To determine the subcellular distribution of TCEAL7 in differentiating myoblasts, we analyzed cytoplasmic and nuclear fractions as a function of time of differentiation. TCEAL7 was detected in both the nucleus and the cytoplasm at the onset of differentiation but became enriched in the nuclear fraction with increasing differentiation time (Fig 2D). The data reinforce the idea that TCEAL7 is found in the nucleus but leaves open the possibility of both cytoplasmic and nuclear functions for TCEAL7.

### The *Tceal7* promoter is inducibly bound by BRG1 during differentiation

Work from others has demonstrated that myogenic regulatory factors (MRFs) activate *Tceal7* expression via E box elements, which are MRF binding sites, in the *Tceal7* promoter [47,48]. As BRG1-based mSWI/SNF complexes cooperate with MRFs to activate myogenic gene expression [27,28,68], we predicted that BRG1 should be bound to the *Tceal7* promoter when it is activated. BRG1 ChIP assays showed inducible binding to the *Tceal7* promoter that increased as a function of time of differentiation (Fig 2E). BRG1 binding correlated with the increase in mRNA as well as with the timing of BRG1 binding to the *myogenin* promoter, a known BRG1 target [27,28,68]; (Fig 2F). Together, these data establish *Tceal7* as a differentiation-induced gene that is likely directly regulated by BRG1.

### Calcineurin A regulates *Tceal7* expression and myoblast fusion

**Fig 3:**
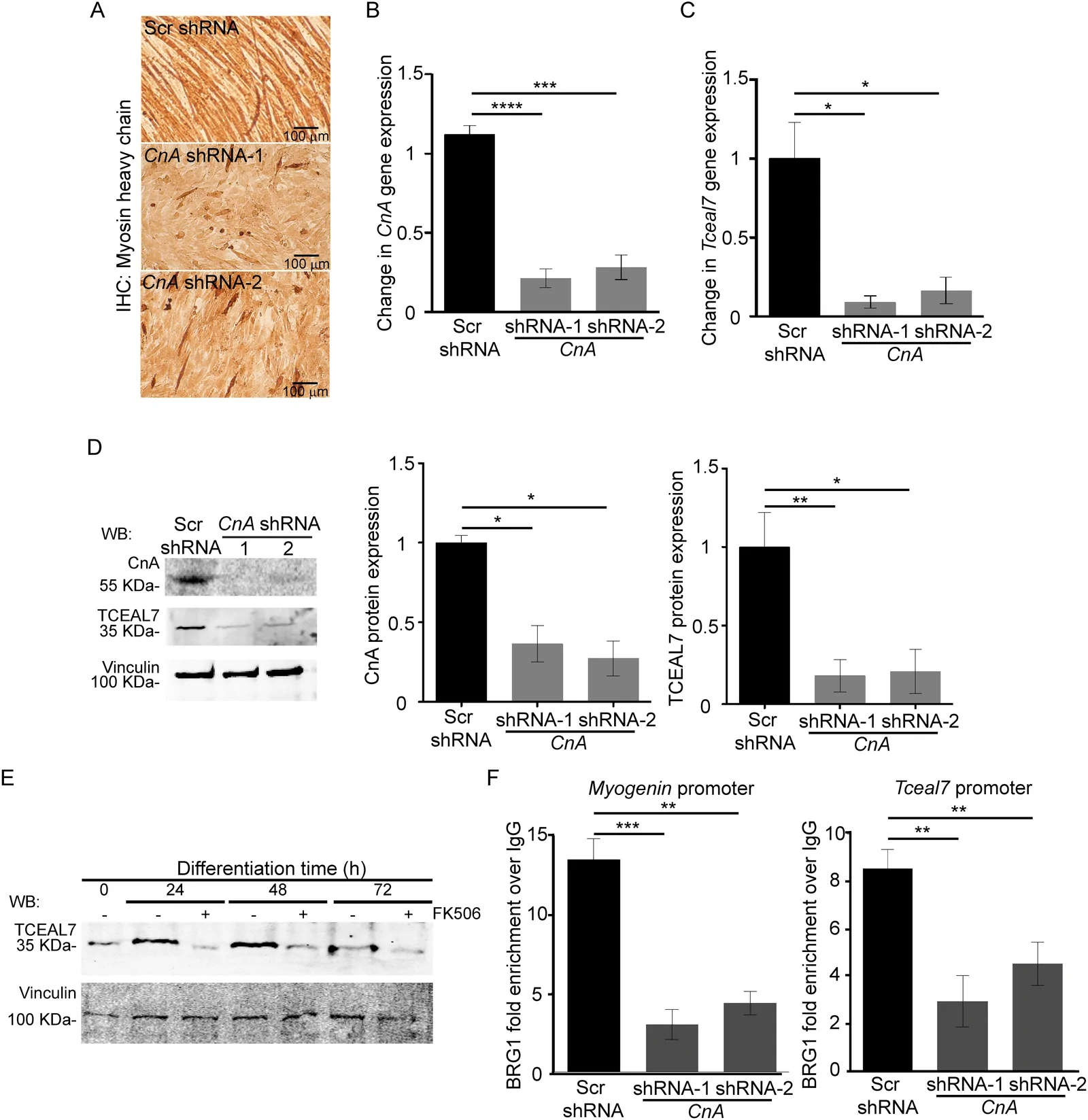
Calcineurin-A knockdown or inhibition impairs myoblast differentiation and suppresses *Tceal7* expression. **(A)** Representative myosin heavy chain immunostaining of 72 h differentiated C2C12 myoblasts transfected with control scrambled sequence (Scr) or one of two distinct siRNAs targeting *CnA*. **(B)** Steady state mRNA expression of *CnA* in 72 h differentiated Scr control and *CnA* knockdown C2C12 myoblasts confirming the efficacy of the *CnA* knockdown. **(C)** Steady state mRNA expression of *Tceal7* at 72 h of differentiation in Scr control and *CnA* knockdown myoblasts. mRNA expression data was normalized to *Eef1a*. **(D)** Representative western blot confirming *CnA* knockdown and reduced expression of TCEAL7 in 72 h differentiated C2C12 myoblasts. Vinculin was used as the loading control. **(E)** Western blot showing TCEAL7 protein levels in FK506-treated C2C12 cells at the indicated differentiation time points. Vinculin was used as the loading control. **(F)** Chromatin immunoprecipitation showing BRG1 occupancy at the *Myogenin* and *Tceal7* promoters; values were normalized to values obtained for an IgG ChIP. (F) Data represent 3 independent biological experiments ± SD. Two-tailed Welch’s t-test statistical analyses. *P < 0.05; **P < 0.01; ***P < 0.001; ****P < 0.0001.

Since BRG1 cooperates with calcineurin (Cn) to activate myogenic gene expression and promote myoblast differentiation [57,69], we next tested whether this phosphatase also contributes to the regulation of *Tceal7*. We generated myoblasts using shRNA targeting *CnA* (the A subunit of the Cn protein; [70]) and used immunohistochemistry analyses of differentiating myoblasts stained against myosin heavy chain to confirm that *CnA* knockdown disrupted myotube formation, producing thinner and poorly fused myotubes relative to controls (Fig 3A). Q-PCR analysis of these cells confirmed knockdown of *CnA* (Fig 3B). *CnA* knockdown reduced the levels of *Tceal7* mRNA (Fig 3C). Immunoblotting showed that CnA and TCEAL7 protein levels (Fig 3D) were also reduced. Together, these results show that Cn signaling is required for proper activation of *Tceal7* during myoblast differentiation. To demonstrate that CnA activity modulates TCEAL7 protein abundance, we treated differentiating C2C12 cells with the Cn inhibitor FK506 [71]. TCEAL7 protein levels decreased across differentiation time points in FK506-treated cultures (Fig 3E), confirming that Cn activity is necessary to sustain *Tceal7* expression. Because BRG1 function is sensitive to CnA activity [69], we examined the binding of BRG1 to the *Tceal7* promoter as well as to the promoter driving the expression of the myogenic marker gene, *Myogenin*, which we have previously shown is sensitive to *CnA* inhibition [69]. BRG1 binding to both promoters was reduced in the presence of shRNA targeting CnA (Fig 3F). These results reveal that Cn signaling promotes *Tceal7* expression at least in part by facilitating BRG1 recruitment to the *Tceal7* promoter.

### *Tceal7* is required for myoblast differentiation and regulated myoblast proliferation

**Fig 4:**
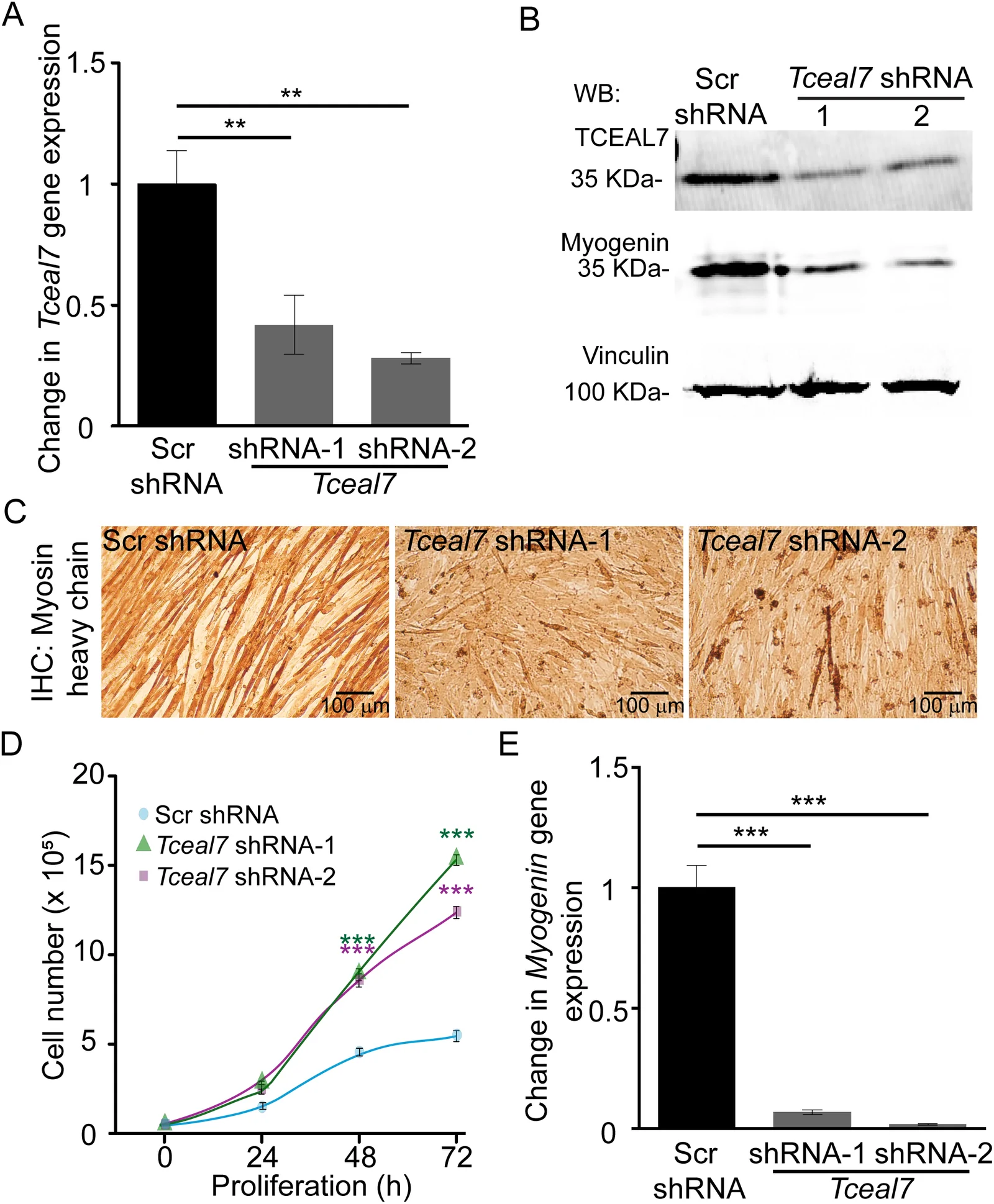
*TCEAL7* KD compromises C2C12 myogenic differentiation. **(A)** Steady state mRNA expression levels of *Tceal7* at 72 h of differentiation; the data were normalized to *Eef1a* expression. **(B)** Representative western blot validating siRNA-mediated *Tceal7* knockdown and showing a corresponding decrease in Myogenin expression in 72 h differentiated C2C12 myoblasts. Vinculin was used as loading control. **(C)** Representative light microscopy analyses of myosin heavy chain staining of differentiated C2C12 cells (72 h) transfected with Scr control or *Tceal7* siRNA. **(D)** Cell proliferation analysis comparing *Tceal7* knockdown myoblasts maintained in proliferation conditions with the Scr control myoblasts. **(E)** Steady state mRNA expression levels for *Myogenin* at 72 h differentiation; data were normalized to *Eef1a* expression. Data represent 3 independent biological experiments ± SD. Two-tailed Welch’s t-test statistical analyses. ** P < 0.01; ***P < 0.001.

Given the differentiation-dependent increase in *Tceal7*, we investigated its functional requirement in myoblast differentiation. shRNA-mediated reduction of *Tceal7* gene expression and protein levels were confirmed by steady state qPCR (Fig 4A) and western blot (Fig 4B). Myoblasts with *Tceal7* knockdown failed to differentiate as demonstrated by reduced MHC staining and the lack of myotubes (Fig 4C). Prior work demonstrated that *Tceal7* overexpression in C2C12 cells decreased cellular proliferation and increased differentiation capacity [47]. Knockdown of *Tceal7* increased myoblast proliferation (Fig 4D), consistent with prior results. Knockdown of *Tceal7* also reduced Myogenin protein (Fig 4B) and *myogenin* mRNA (Fig 4E) levels, consistent with a requirement for differentiation. TCEAL7 therefore likely contributes to myoblast differentiation by contributing to both cell cycle regulation and to the induction of myogenic gene expression.

### Transcriptomic profiling reveals that TCEAL7 controls differentiation and transcription factor networks

**Fig 5:**
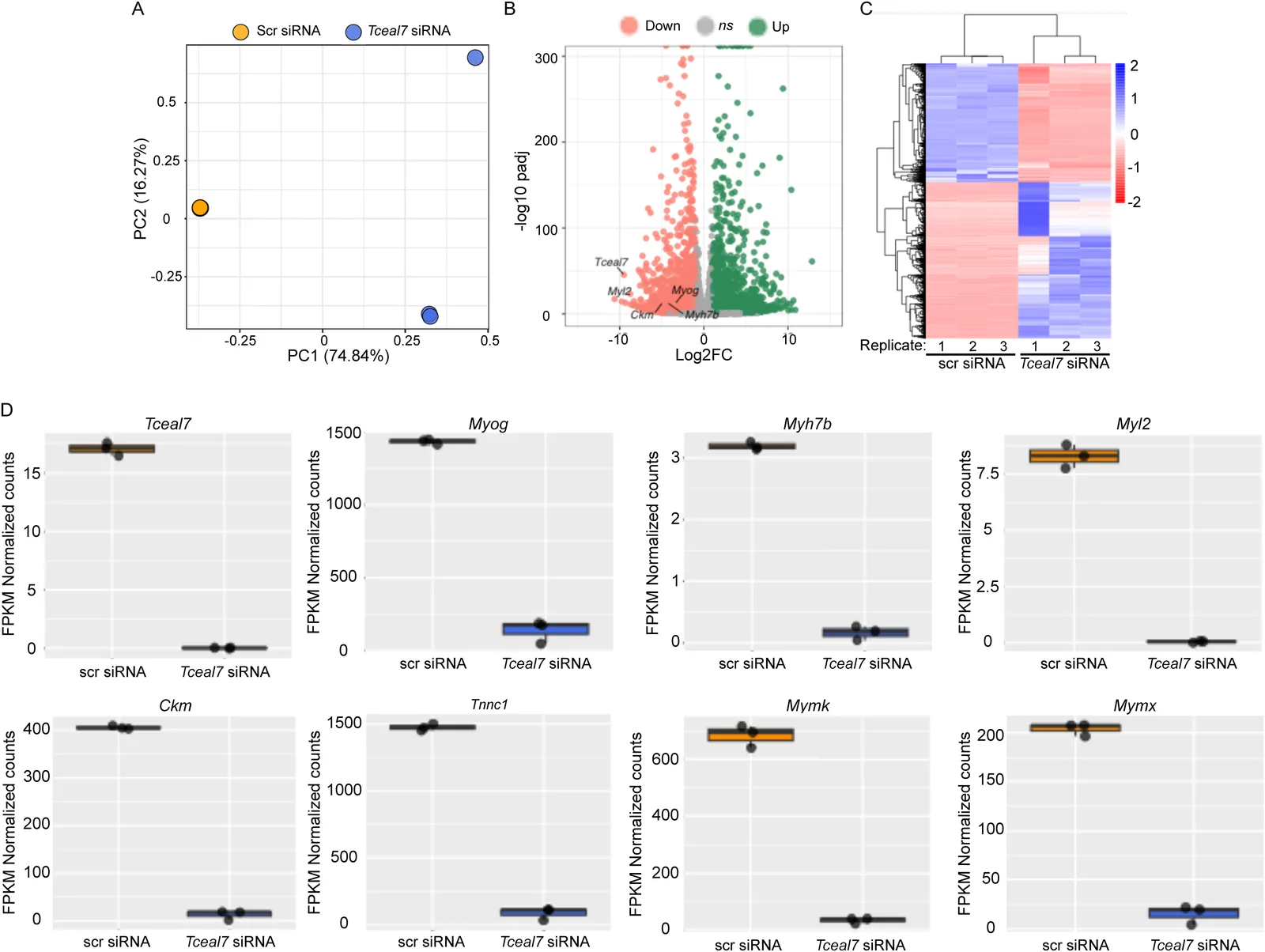
RNA sequencing of differentiated *Tceal7* knockdown C2C12 myoblasts shows suppression of myogenic genes. **(A)** PCA plots showing log_2_FPKM normalized counts from three biological replicates for both Scrambled-sequence shRNA and *Tceal7* knockdown samples. **(B)** Volcano plot of significantly dysregulated genes. *Tceal7* and representative myogenic genes are labeled. **(C)** Heatmap of dysregulated genes with hierarchical clustering shows difference in expression patterns between biological groups and replicates. **(D)** Boxplots of normalized FPKM counts for selected myogenic genes are shown. Sh_*Scr*, scrambled-sequence shRNA samples; Sh_*Tceal7*, *Tceal7* shRNA samples.

To define the global transcriptional programs regulated by *Tceal7*, we performed RNA-seq on three independently generated differentiating *Tceal7* knockdown C2C12 myoblasts. Scr control and *Tceal7* knockdown myoblasts were induced to differentiate for 72 h, and total RNA was extracted and processed for RNA-seq analyses. Principal component analysis (PCA) of the log_2_ FPKM normalized counts from the datasets obtained is shown in Fig 5A. Fig 5B shows a volcano plot of the significantly up- and down-regulated genes in *Tceal7* knockdown cells. A heatmap of dysregulated genes further demonstrates the global changes in gene expression due to *Tceal7* knockdown (Fig 5C). Representative myogenic genes that were down-regulated by *Tceal7* knockdown are labeled in Fig 5B and boxplots highlighting the changes in the representation of these genes is shown in Fig 5D.

**Fig 6:**
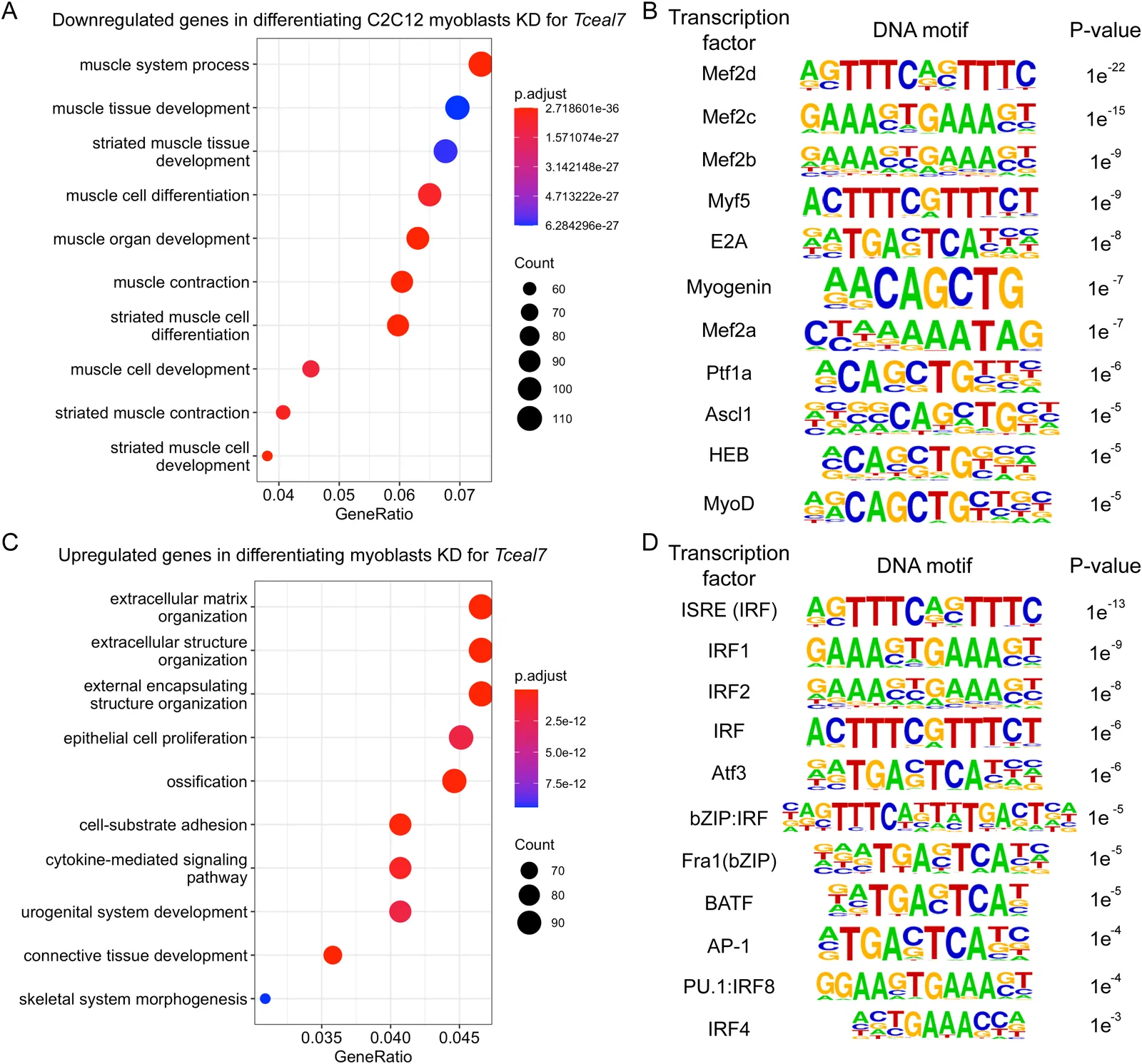
Transcriptomic profiling of differentiated *Tceal7* knockdown C2C12 myoblasts revealed dysregulation of differentiation and cell cycle programs. GO analysis of downregulated (**A**) and upregulated (**C**) genes in differentiated *Tceal7* knockdown myoblasts compared to differentiated Scr-transfected control myoblasts. HOMER known-motif enrichment for downregulated **(B)** and upregulated (**D**) genes.

The differentially expressed genes (DEGs) are listed in S2 Table. Gene Ontology (GO) analysis showed that the top ten categories of downregulated genes were all biological processes associated with muscle development, differentiation, and/or function (Fig 6A). Motif enrichment analysis revealed that downregulated genes were highly enriched for motifs associated with several myogenic transcription factors (Fig 6B). The motifs associated with downregulated genes were binding sites for the MRFs, MyoD1, Myf5, and myogenin, and for the E proteins E2A and HEB that heterodimerize with MRFs [53,72]. These results indicate suppression of the core myogenic regulatory protein network that drives myoblast commitment, differentiation, and fusion. Down regulated genes are also significantly enriched for binding sites for MEF2 family factors (Mef2a, Mef2b, Mef2c, Mef2d), which are cellular transcription factors that augment MRFs to promote transcriptional programs required for sarcomere assembly, metabolic maturation, and myofiber growth [73]. The motifs common to genes that were down regulated by *Tceal7* knockdown reinforce the conclusion that *Tceal7* broadly regulates the myogenic transcription program.

Upregulated genes were enriched for extracellular matrix structure and organization and cell adhesion. Proliferation and differentiation processes related to other cell types were also identified (Fig 6C). Motif analyses of overexpressed genes (Fig 6D) showed a significant enrichment of ISRE/IRF, IRF1, IRF2, IRF4, and PU.1:IRF8 motifs, suggesting strong activation of interferon-responsive and innate immune transcriptional programs [74]. Identification of motifs for ATF3, BATF, Fra1, and other bZIP/AP-1 factors further point to enhanced stress, cytokine, and inflammatory signaling. Together, the motif profile suggests that reduction of *Tceal7* expression shifts myoblasts toward a heightened immune-stress transcriptional state that is incompatible with effective myogenic differentiation.

**Fig 7:**
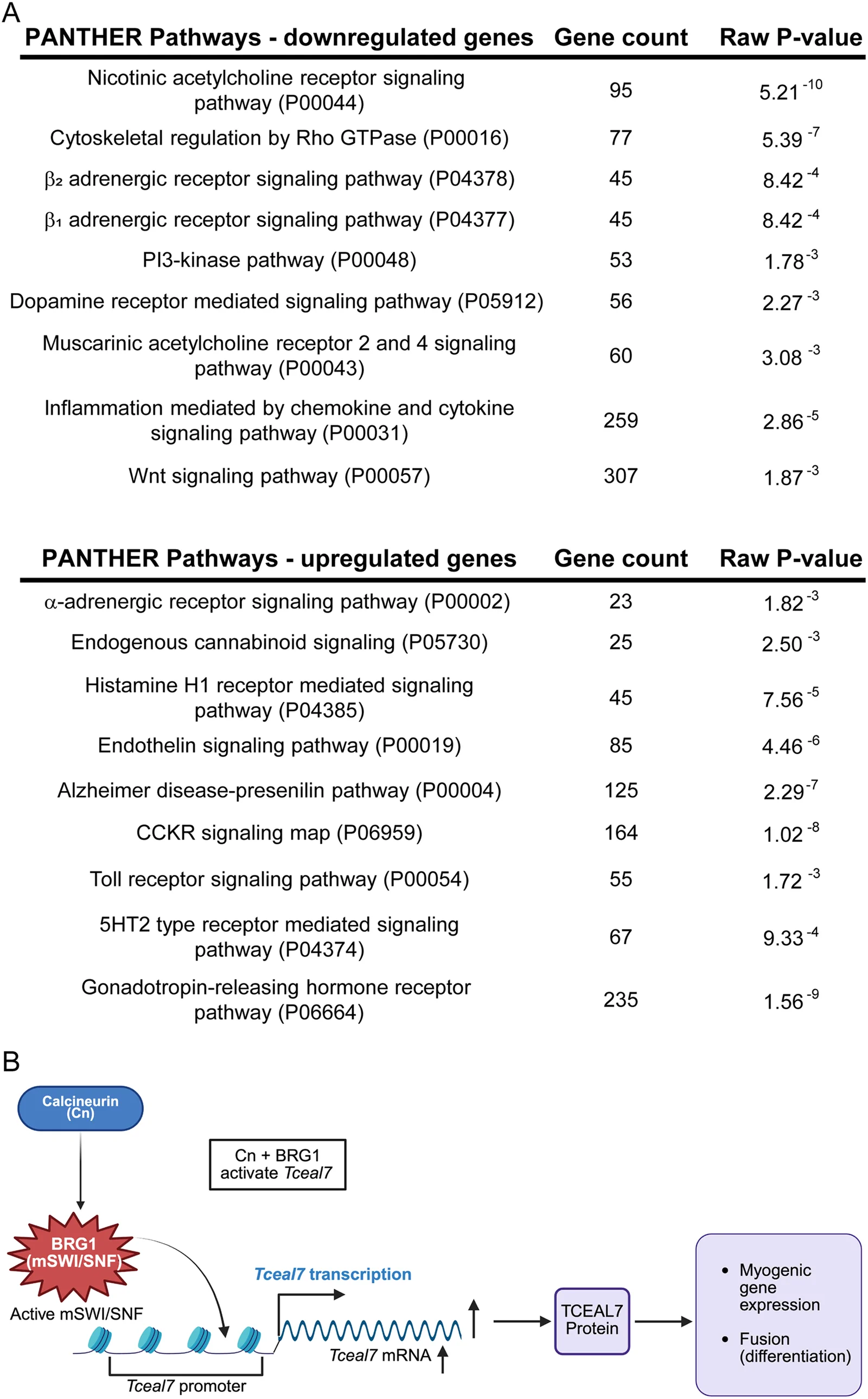
PANTHER analysis and schematic model. **(A)** Top terms for downregulated and upregulated genes in differentiated *Tceal7* knockdown myoblasts. **(B)** Schematic model for Cn- and BRG1-dependent activation of *Tceal7* gene expression during myoblast differentiation. Figure created with Biorender.

PANTHER classification further identified structural and regulatory proteins affected by *Tceal7* knockdown. Among the top categories associated with downregulated genes were pathways that are linked to skeletal muscle development and function (Fig 7A). For instance, the most affected pathway was nicotinic acetylcholine receptor signaling, with 95 genes downregulated. This pathway is essential for neuromuscular junction formation and activity-dependent maturation of myofibers, promoting terminal differentiation and sarcomere organization [75]. Seventy-seven genes related to cytoskeletal regulation by Rho GTPases, which controls actin dynamics, myoblast shape, migration, and the membrane remodeling required for myoblast alignment and fusion were repressed by *Tceal7* knockdown. Chemokine- and cytokine-mediated inflammation pathways showed decreased expression of 259 related genes. These inflammatory pathways are known to regulate the inflammatory milieu required for proper muscle regeneration, balancing early pro-inflammatory cues that promote myoblast activation with later anti-inflammatory signals that enable differentiation and fusion [76]. β_1-_ and β_2-_adrenergic receptor signaling, which broadly impacts muscle and muscle stem cell function [77–79], were also significantly affected with 45 genes downregulated. The PI3K pathway is a central driver of myogenesis, integrating growth factor cues to stimulate Akt signaling, protein synthesis, survival, and myotube growth [80]. Fifty-three genes associated with PI3K were repressed. Finally, the Wnt signaling pathway, with 307 genes downregulated, represented the largest number of genes altered. The Wnt pathway is a well-established regulator of skeletal myogenesis, directing progenitor cell fate, initiating differentiation programs, and coordinating myotube formation and growth [41,42,81].

PANTHER analyses also provided insight into upregulated pathways, identifying signaling programs that may impact skeletal muscle development, regeneration or function either directly or indirectly (Fig 7B). Among these was endothelin receptor signaling, recently shown to inhibit myoblast proliferation and MRF and other myogenic gene expression through the p38 MAPK pathway. Chronic exposure to endothelin led to skeletal muscle atrophy and poor exercise performance [82], Endothelin also promotes muscle fibrosis [83]. α-adrenergic receptor, a G-protein coupled receptor best known for its role in vasoconstriction, indirectly affects muscle function [84] but may have direct effects as well, as chemical mimics of pathway components resulted in depletion of a histone deacetylase from the nucleus of slow-twitch soleus muscle fibers, resulting in altered gene regulation [85]. Endogenous cannabinoid signaling uses lipid messenger molecules to regulate numerous body functions. Levels of an endogenous cannabinoid normally decrease during satellite cell and myoblast differentiation, and its continued presence inhibits differentiation [86]. Thus, upregulation of this pathway by *Tceal7* knockdown correlates well with the observed inhibition of myoblast differentiation observed upon *Tceal7* knockdown. Finally, presenilin knockdown led to accelerated myoblast differentiation during muscle regeneration in vivo, while constitutive presenilin expression suppressed differentiation [87]. Collectively, the PANTHER analysis confirms the widespread impact of *Tceal7* knockdown on the fidelity of gene expression in myoblasts, with broad effects across a spectrum of genes and signaling pathways.

## Discussion

Collectively, these results identify *Tceal7* as a BRG1-dependent, calcineurin-responsive regulator of myogenic differentiation. *Tceal7* is induced during differentiation, localizes to both the nucleus and the cytoplasm, and is required for myogenin expression, myotube fusion, and activation of myogenic gene networks. *Tceal7* knockdown also upregulates GPCR-, cytokine-, and interferon-related pathways and IRF/AP-1 responsive-genes, shifting cells toward an inflammatory stress state that are likely incompatible with differentiation. Our results support a model in which calcineurin signaling promotes BRG1 recruitment to the *Tceal7* promoter, enabling its expression and activation of the downstream transcriptional environment required for efficient myogenic commitment and skeletal muscle formation, maturation, and function (Fig 7C).

Identification of TCEAL7 as a nuclear protein is consistent with a report of ectopically expressed TCEAL7 being found in the nucleus [66]. BRG1 and mSWI/SNF enzymes are predominantly reported as nuclear proteins [34,88], which supports the idea of a direct mechanism of BRG1-mediated activation of *Tceal7* expression. Conclusive identification of cytoplasmic TCEAL7 is, to our knowledge, a novel observation. Additional work would be required to determine whether there are specific cytoplasmic functions for TCEAL7 or whether cytoplasmic sequestration is a mechanism to regulate the amount of nuclear protein. Since CnA fits into the process of activation of TCEAL7-dependent gene expression and since CnA dephosphorylates BRG1 as part of the activation process [69], an intriguing possibility is that CnA also regulates TCEAL7 via regulation of its phosphorylation state.

The TCEAL gene family comprises a cluster of X-linked genes conserved across mammals. Members of this family were initially presumed to function as transcription elongation factors based on early reports that TCEAL1 shared limited homology with TFIIS, an RNA polymerase II elongation factor containing a characteristic Zn-binding domain [3]. However, structural comparisons using AlphaFold2 have since demonstrated that TCEAL proteins lack the Zn-binding and Pol II-interacting domains of TFIIS, and no biochemical evidence supports their direct participation in transcriptional elongation or RNA polymerase II binding [10]. Thus, the molecular activities of TCEAL proteins remain largely undefined. It remains possible that TCEAL7 plays a direct role in regulating transcription. It is also possible that it indirectly affects gene expression, perhaps through the de-ubiquitination function identified in other family members [11–13] or in a yet undiscovered function. A more general and indirect mechanism of action may make sense, given the extensive effect seen in the analyses of the RNA-seq data from the *Tceal7* knockdown myoblasts where genes associated with a multitude of muscle and muscle-related functions and pathways were mis-regulated. The wide-spread association between mis-regulation of genes encoding TCEAL family members with a large number of disparate types of cancers [10] may also argue that TCEAL7 and other family members have a general and indirect impact on gene expression.

Our results indicate that knockdown of *Tceal7* inhibits myoblast differentiation and myogenic gene expression. A prior report [46] indicated that a combination of *Tceal7* and *Tceal5* knockdown was needed to negatively impact myoblast differentiation. That work demonstrated robust knockdown of the targeted mRNAs but did not measure protein expression. It is possible that our *Tceal7* knockdown vector was more efficient at reducing the amount of TCEAL7 protein, thereby negating the need for concurrent *Tceal5* knockdown. This scenario predicts that efficient knockdown of *Tceal5* would also inhibit myoblast differentiation, which is a testable hypothesis for future studies.

The regulation of the *Tceal7* gene during embryonic development and during in vitro myoblast differentiation established that MyoD and related MRFs, along with cooperating factors, regulate transcription in muscle [46–50]. Our work extends these findings to demonstrate that the mSWI/SNF chromatin remodeling enzymes containing BRG1 as the ATPase subunit also regulate the expression of *Tceal7*. We have previously shown that BRG1 remodels chromatin at target genes to facilitate stable binding of MRFs [28]; it is likely that this function extends to the *Tceal7* promoter. In addition, we demonstrated that BRG1 function is sensitive to chemical inhibition of reduction in expression of the Cn phosphatase and demonstrated that dephosphorylation of BRG1 was associated with its activation at target promoters [69]. In keeping with the prior model, we propose that Cn-dependent modification of BRG1 promotes stable binding of MRFs at the *Tceal7* promoter, thereby facilitating gene induction during myoblast differentiation. It remains to be seen whether regulation of *Tceal7* gene expression by Cn and BRG1 regulation occurs in other cell types. Another interesting question is whether Cn and BRG1 mediate regulation of the genes encoding other TCEAL family members.

## Conclusions

In summary, this study identifies TCEAL7 as a critical downstream effector of BRG1-dependent chromatin remodeling and calcineurin signaling during myoblast differentiation. We propose a model in which *Tceal7* expression is facilitated by BRG1 recruitment to the *Tceal7* promoter, where its chromatin remodeling activity is activated by calcineurin phosphatase, resulting in the stable association of MRFs and other cooperating transcription factors that drive myogenic gene expression. We also conclude that TCEAL7 plays an essential role in coordinating signaling pathways, cell cycle exit, the induction of the myogenic transcription programs, and efficient myoblast fusion into myotubes. Moreover, our work demonstrates that the spectrum of TCEAL7 function is extremely broad, impacting nearly every aspect of myoblast differentiation. Additionally, we suggest that TCEAL7 function will be generally required for not only for tissue differentiation and development but also for cell proliferation and for cell and tissue homestasis.

## Supporting information

S1 Table Primer sequences used in this study. (PDF)

S2 Table List of differentially expressed genes (DEGs). (XLSX)
