# Supplementary material for: Tceal7 is a BRG1-regulated target of calcineurin signaling that promotes myoblast differentiation
Source: PLoS One. 2026 Sep 16;21(9):e0358038. doi: 10.1371/journal.pone.0358038 (PMC13581019; doi:10.1371/journal.pone.0358038)
Supplement: Supplementary file 1 [file pone.0358038.s001.pdf]

Supplemental Table 1

Primers for qPCR (5’-3’)

Tceal7	FW: ACTTGTGGCCAAGGAGAAGA
	REV : GGGTTGTTCATCCTCCCTCT
CnA	FW: GTTTAAACAAGAATGTAAAATAAA
	REV : GAATCGGTCTAATTTTCTGATGTC
Myogenin	FW: GTCCCAACCCAGGAGATCATTTGCTC
	REV : CCACTTAAAAGCCCCCTGCTA
Eef1A1	FW: GGCTTCACTGCTCAGGTGATTATC
	REV: ACACATGGGCTTGCCAGGGAC

Primers for ChIP (5’-3’)

Tceal7	FW: CCGCCCGAAAGGTCTATATT
	REV: TTTTACCTTTCCCTGATATCTTCC
Myogenin	FW: ACACCAACTGCTGGGTGCCA
	REV: GAATCACATGTAATCCACTGG
